# Understanding the impact of stressors on safety behavior of Chinese special equipment operators: a transactional theory of stress perspective

**DOI:** 10.3389/fpubh.2026.1775181

**Published:** 2026-03-04

**Authors:** Jing Jing Zhang, Jin Cai Zhang, Yang Bai, Xiao Chang Liu, Ran Liu, Ze Shi Yang

**Affiliations:** 1 School of Management Science and Engineering, China Jiliang University, Hangzhou, China; 2 Special Equipment Safety Supervision Inspection Institute of Jiangsu Province, Nanjing, China; 3 China Special Equipment Inspection and Research Institute, Beijing, China; 4 Shanghai Special Equipment Supervision and Inspection Technology Institute, Shanghai, China

**Keywords:** appraisal, coping strategy, safety behavior, special equipment operators, stressors, transactional theory of stress

## Abstract

**Introduction:**

Special equipment operator safety behavior is a critical factor in preventing accidents involving special equipment. To mitigate such incidents, it is essential to understand and address the stress and coping mechanisms of operators. This study, grounded in the transactional theory of stress (TTS), aims to identify the stressors associated with special equipment operators and to examine the relationships between these stressors, cognitive appraisal, coping strategies, and safety behavior.

**Methods:**

This research employed a quantitative method, with data collected through a questionnaire survey, a total 735 validity responses was collected and analyzed using SmartPLS.

**Results:**

The results demonstrate that low social status (LSS), harsh working environments (HWE), physiological fatigue (PF), and responsibility pressure (RP) are significant stressors for special equipment operators. Moreover, the study reveals that emotion-focused coping mediates the relationships between threat appraisal (TA) and safety behavior, as well as between harm appraisal and safety behavior. In addition, problem-focused coping is found to mediate the relationship between challenge appraisal (CA) and safety behavior. Furthermore, government intervention is shown to negatively moderate the relationship between emotion-focused coping and safety behavior.

**Discussion:**

This study contributes to a deeper understanding of the transactional theory of stress and provides practical insights for accident prevention, offering valuable guidance for enhancing occupational safety.

## 1 Introduction

**Figure 1:**
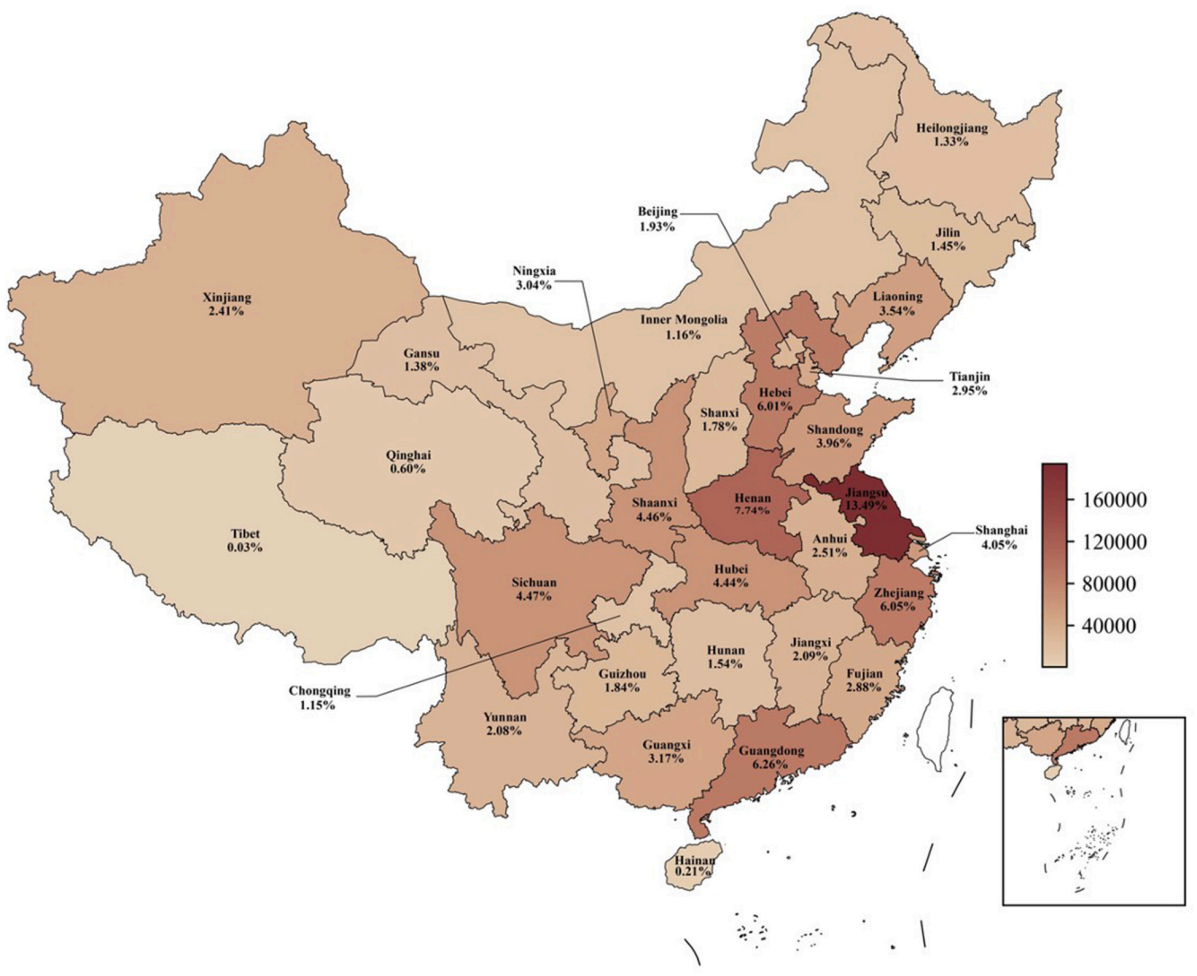
The proportion of special equipment operators in each province.

In China, special equipment^1^ represents a critical symbol of national economic development, encompassing essential machinery and facilities that are vital to both industrial production and daily life. These pieces of equipment are characterized by high-risk factors, a potential for significant damage, and a heightened likelihood of sudden accidents. The manufacturing, installation, testing, and utilization of special equipment inherently involve numerous risks, with accidents often leading to severe consequences. For instance, a gas explosion in Ningxia City in 2023 resulted in 31 fatalities and seven injuries (1). Such incidents not only cause substantial loss of life and property damage but also incite widespread social panic. Besides, although the Chinese government continues to revise and improve relevant laws and regulations, many companies and practitioners still engage in non-compliant operations, which are associated with the occurrence of accidents. Therefore, ensuring the safety of special equipment is imperative and cannot be overlooked. This study aims to address this critical issue by providing a comprehensive examination of safety behavior related to special equipment, thereby contributing to the broader discourse on industrial safety and risk management. Figure 1 illustrates a map of China, displaying the proportion of special equipment operators in each province as of April 2024, and the operators are concentrated in central and coastal cities.

Accidents involving special equipment have been a persistent issue, consistently drawing attention from both the government and society. Data on special equipment accidents released by authorities over the years indicates that these incidents are primarily attributed to two factors: unsafe human behavior and equipment malfunctions, with unsafe behavior by operators^2^ accounting for approximately 80% of all accidents annually (2). This result indicated that special equipment operators' unsafety behavior is the main reason that led to the accident. Such result is consistence with Derdowski and Mathisen (3), that human-factor causes can be attributed to accidents in high-risk industries. Occupational safety and health problems have become a major problem to be faced and solved in the modern industrial countries all over the world, especially in China (4). Individuals engaged in high-risk work are particularly susceptible to stress, which significantly affects their safety behaviors (5). This suggests that accident prevention efforts should focus primarily on addressing the stress experienced by operators, which is associated with their safety behavior. Existing literature predominantly addresses special equipment accident causes related to equipment failures (6, 7), leaving a significant gap in research concerning special equipment operators' safety behavior. This highlights the need for further investigation into special equipment operators' safety behaviors to enhance the understanding and prevention of accidents in this critical area. Therefore, this study aims to fill this research gap by exploring various stressors [e.g., responsibility pressure (RP), harsh work environment (HWE), low social status (LSS) and physiological fatigue (PF)] that contribute to operators' safety behavior as a means of preventing accidents.

Besides, accidents often result from a complex interaction of cognitive, emotional, and environmental factors, with stress playing a significant role (8). To enhance safety, it is crucial to understand how stressors are associated with safety behavior through cognitive appraisal and coping mechanisms (9). The transactional theory of stress (TTS) is employed as the theoretical foundation in this study, as it provides a robust framework for understanding the generation, perception, and coping mechanisms associated with individual stress (10, 11). This aligns well with the objectives of this paper. While TTS has been extensively applied in research on technostress, there has been limited exploration of the appraisal and coping processes (12), particularly in the context of special equipment operations. Therefore, investigating the appraisal and coping processes of special equipment operators within the framework of TTS is especially valuable.

Based on the above, this study will focus on addressing the following two research questions:

1. What are the key potential stressors of special equipment operators?
2. What are the mechanisms through which stressors are associated with special equipment operators' safety behavior, based on the transactional theory of stress?

## 2 Literature review

### 2.1 Transactional theory of stress

Transactional Theory of Stress (TTS), proposed by Lazarus and Folkman (13), is a widely recognized framework for understanding how individuals perceive and respond to stress. The model posits that stress does not arise directly from external stimuli, but rather from an individual's cognitive appraisal of those stimuli and their perceived ability to cope (10). According to this theory, when an individual encounters a potential stressor, they first engage in primary appraisal, evaluating whether the event poses a threat, harm, or challenge.

In addition to primary appraisal, TTS highlights the concept of secondary appraisal, which refers to an individual's evaluation of available resources and capacity to cope with a stressor (13). While primary appraisal determines whether a stressor is perceived as threatening, challenging, or harmful, secondary appraisal influences the selection of coping strategies. In the context of this study, secondary appraisal is implicitly reflected in the adoption of problem-focused and emotion-focused coping strategies by special equipment operators. That is, operators evaluate their capacity and resources to manage stressors such as low social status, harsh working environments, physiological fatigue, and responsibility pressure, and this evaluation guides their choice of coping responses.

The primary appraisal refers to an individual's assessment of whether the event is a threat, harm or challenge (10). Threat appraisal (TA) occurs when the situation is perceived as potentially harmful, posing risks to wellbeing or performance, leading to stress or anxiety, the scale usually covers perceived severity (14). An operator might fear the possibility of accidents or mistakes when using or repair the hazardous equipment (e.g., ropeway). Harm appraisal involves the recognition that damage has already occurred, whether physical, emotional, or in terms of performance (13). Challenge appraisal (CA) arises when individuals view a situation as an opportunity for growth or achievement, motivating them to engage positively (15). For example, an operator might see handling complex equipment as a chance to develop technical skills.

In response to appraisal outcomes, individuals may adopt various coping strategies, resulting in differential performance outcomes. Problem-focused and emotion-focused coping strategies are typical coping types (12). Problem-focused coping involves the belief that the stressor is controllable and that, through concerted effort, individuals can alter an environment that is perceived as challenging. Emotion-focused coping refers to the process of alleviating negative emotions through cognitive strategies such as avoidance, distancing, or identifying positive value in negative events, allowing individuals to reframe their interpretation of the stressor without altering the objective situation (13).

Outcome is result of the interaction between the individual, the stressor, and the coping mechanisms (11). In this study, outcome is regarded as safety behavior, which is the result after the process of operators coping the appraisal from stressor.

**Figure 2:**
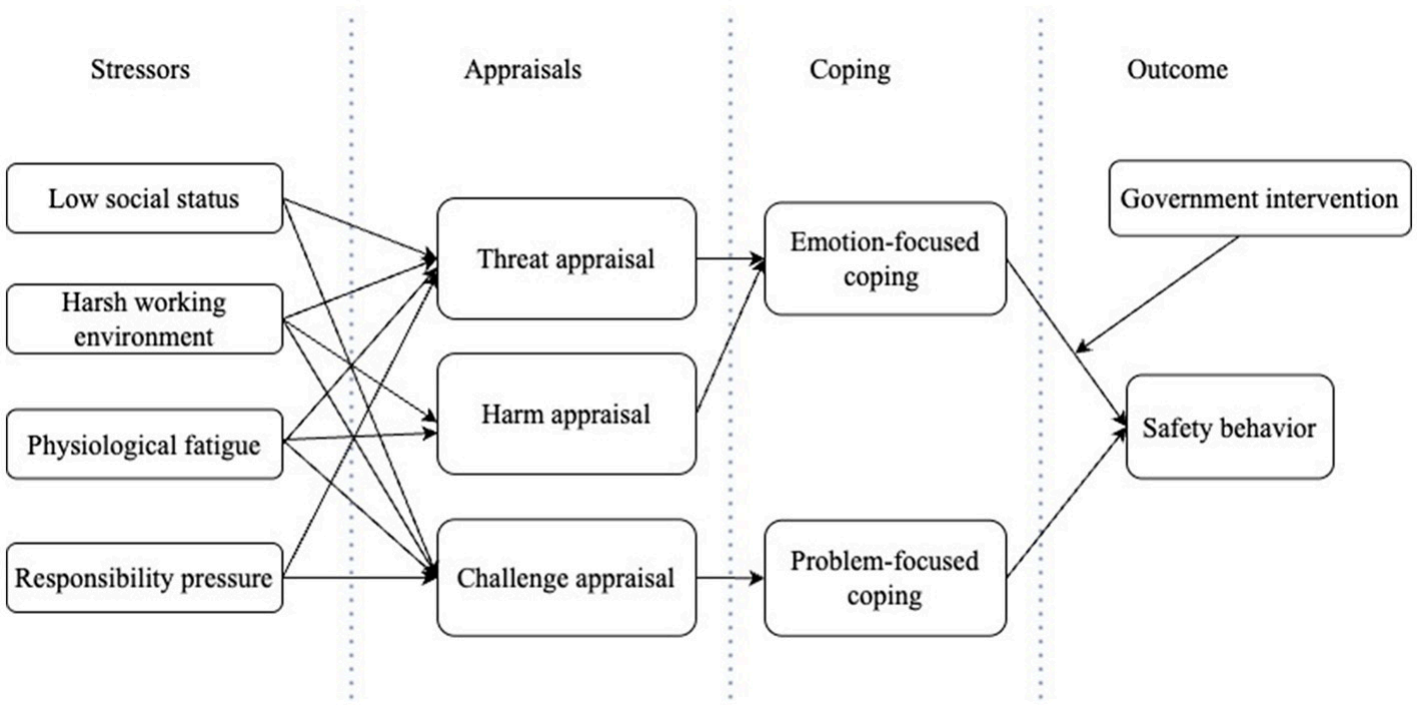
Framework.

In recent years, TTS has been widely applied in analyzing the behavior of high-risk occupational groups (16). The theory seeks to explain how stressors are linked to an individual physiological, psychological, and behavioral responses, and how these responses, in turn, are related to their overall behavior. This aligns with the purpose of the present study, which aims to identify the mechanisms through which stressors associated with the safety behavior of special equipment operators. Drawing on prior research, this study posits that stressors experienced by special equipment operators, to explore how the stressors (low social status, harsh work environment, physiological fatigue and responsibility pressure) are related to their safety behavior through two key mechanisms of appraisal (including threat appraisal and challenge appraisal and harm appraisal) and coping (including emotion-focused and problem-focused coping strategies). Figure 2 presents the conceptual framework of this study.

### 2.2 Low social status and appraisal (threat and challenge)

Despite the vital role of special equipment and its operators across various sectors, such as the chemical, oil and gas, power, and entertainment industries, these workers often face low social status and limited societal recognition. In China, the educational attainment of special equipment operators is generally low, with many relying primarily on manual labor to earn a living (17). Compared to white-collar professionals, they tend to occupy a lower social position. This lack of recognition can result in feelings of isolation and frustration, which have been identified as occupational stressors (18, 19).

From a cognitive appraisal perspective, however, low social status represents a relatively stable and enduring condition rather than an acute or episodic demand. According to the transactional theory of stress, stressors that are persistent and normalized may not consistently elicit strong threat appraisals, as individuals gradually adapt to their circumstances and recalibrate expectations. In this sense, low social status may not be immediately or uniformly appraised as threatening, particularly when individuals perceive limited alternatives or accept their social position as relatively fixed. Over time, however, the persistent absence of validation and societal acknowledgment can erode emotional wellbeing, contributing to chronic strain rather than acute threat responses (20).

Nevertheless, prior research suggests that low social status can elicit threat appraisals under certain conditions, particularly when it heightens perceptions of uncertainty, uncontrollability, or potential loss. In the context of special equipment operators in China, low entry requirements and high replaceability may increase concerns about job security and future prospects. Supporting this view, Scheepers and Ellemers (21) demonstrated that individuals assigned to low-status positions exhibited physiological threat responses when evaluating their subordinate standing during motivated performance tasks. However, such threat responses are likely contingent on situational cues and individual differences, rather than being a uniform consequence of low social status.

While challenge appraisals are generally linked to positive outcomes such as increased engagement and better performance (22), in the context of China's large population and rapidly aging workforce, improving the social status of special equipment operators is particularly difficult. Given the structural constraints and limited upward mobility, these workers may find it challenging to reframe stressors as opportunities for personal growth or achievement. Consequently, status inequality may inhibit proactive, growth-oriented interpretations of job demands, thereby reducing the likelihood of forming challenge appraisals. Based on above discussion, we formulate the following hypotheses:

*H1. Low social status is positively associated with threat appraisal*
*H2. Low social status is negatively associated with challenge appraisal*

### 2.3 Harsh working environment and appraisal (threat, harm, and challenge)

The harsh working environment refers to those outer environmental conditions (e.g., the very warm and extremely cold climates) that are difficult for people to work in (23). It has been widely recognized as a key factor influencing workers' safe and unsafe behaviors (24). The special equipment sector is characterized by a high incidence of occupational accidents and challenging working conditions, such as poor lighting, extreme temperatures, and exposure to hazardous materials, which pose substantial difficulties for operators (25). For example, pressure vessel operators frequently work in extreme environmental conditions, such as high-temperature settings (e.g., boiler rooms) or low-temperature zones (e.g., areas surrounding liquefied gas storage tanks). In some cases, they are required to perform inspections or maintenance tasks within confined spaces, such as the interior of storage tanks. Current study proved that number of accidents in the workplace increase due to the workers' negative emotion (e.g., irritation, anger) that are triggered by the uncomfortable environment (26). Also, the working environment has been consistently identified as a major contributor to task-related stressors across diverse occupational contexts (27, 28). Thus, harsh working environment was considered as one of the stressors in this study.

Besides, from the perspective of the TTS (13), such harsh environments can be subjectively appraised by operators in different ways. Specifically, they may be perceived as threat stressors, due to concerns about potential future health issues, such as the extreme cold environment can decrease job performance, also occurs when the core body temperature falls significantly (26). Harm stressors, as a result of psychological trauma stemming from past accidents or injuries, like the fall accident caused by workers repairing amusement facilities at high altitude. Also, challenge stressors, when individuals interpret the environment as an opportunity to improve their skills and resilience. This view is consistent with findings by Liang (29), who suggest that under certain complex conditions, employees may exhibit greater creativity and proactive behavior when confronting stress. Based on above discussion, we formulate the following hypotheses:

*H3. Harsh working environment a is positively associated with threat appraisal*
*H4. Harsh working environment a is positively associated with harm appraisal*
*H5. Harsh working environment a is positively associated with challenge appraisal*

### 2.4 Physiological fatigue and appraisal (threat, harm, and challenge)

Physiological fatigue is a prevalent condition among special equipment operators due to long working hours, repetitive tasks, and physically demanding activities, which can compromise safe and efficient performance (30). Operators frequently work in extreme or variable outdoor conditions, such as intense heat, cold, heavy rain, snow, or strong winds, further exacerbating fatigue. For instance, forklift operators often work up to 12 h per day, while boiler workers may need to install or repair equipment outdoors under harsh climatic conditions. Despite its traditional conceptualization as an outcome of stress, physiological fatigue can also function as a stressor in high-risk occupational contexts. According to the Transactional Theory of Stress (TTS) (13), stress arises from the interaction between environmental demands and individual coping resources. Within this framework, fatigue represents a salient situational strain that can impair cognitive appraisal and diminish coping efficacy. This conceptualization aligns with the Job Demands–Resources model, which posits that sustained job demands can lead to fatigue, which in turn shapes appraisal and behavior (20, 31, 32). Occupational health authorities also recognize fatigue as a critical factor influencing performance and safety outcomes (33). Thus, this study identifies physiological fatigue as a key stressor and seeks to investigate its association with the safety behavior of special equipment operators.

Besides, when fatigue becomes overwhelming, it tends to elicit operators' threat and harm appraisals. Operator may feel the extreme physiological fatigue will cause occupational diseases, such as cervical spondylosis, cardiovascular disease. Also, Xing et al. (34) indicated that the physiological fatigue can induce the mental fatigue status and other mental performances of the brain. It means that the harm (mental fatigue) from physiological fatigue already occurred. Similarly, for special equipment operators, it also can be explained that physiological fatigue may arise their harm appraisal. Moreover, fatigue exacerbates stress when operators are expected to maintain high performance despite diminishing energy and concentration levels (35). In such conditions, challenge appraisals, which involve perceiving a situation as an opportunity for growth or mastery, may also be adversely affected, as physical fatigue inhibits the generation of proactive, goal-oriented thoughts and behaviors. This view is supported by Zhang et al. (17), that construction workers' physical fatigue is negatively associated with their cognitive ability and motion ability. Based on above discussion, we formulate the following hypotheses:

*H6. Physiological Fatigue is positively associated with threat appraisal*
*H7. Physiological Fatigue is positively associated with harm appraisal*
*H8. Physiological Fatigue is negatively associated with* challenge *appraisal*

### 2.5 Responsibility pressure and appraisal (threat and challenge)

Job responsibility refers to the extent to which workers are held accountable for the outcomes of their work (36). Integrating this concept with the definition of stress, this study defines responsibility pressure as the psychological burden arising from being accountable for the safe operation of complex machinery and the wellbeing of others in the workplace. Special equipment operators often face serious consequences for errors, which may result in accidents or injuries. In China, the insurance mechanism for special equipment accidents remains underdeveloped. Consequently, when such accidents occur, operators may be required to assume part of the resulting liability. For instance, through revocation of their professional qualifications or financial penalties. Therefore, given the complexity of their work environment and the high stakes involved, operators frequently experience considerable stress. Besides, Responsibility has also been identified as a major contributor to task-related stressors across a wide range of occupations (19, 28). Accordingly, this study includes responsibility pressure as a key potential stressor.

For special equipment operators, when responsibility pressure is perceived as overwhelming or likely to result in failure and negative consequences, it may be appraised as a threat. For instance, forklift operators are susceptible to delivering incorrect goods or unintentionally causing harm to others due to operational errors. Motivated by a strong sense of responsibility to ensure smooth workflows and protect the safety of those around them, they often experience considerable stress. However, since such errors are frequently unpredictable and difficult to fully control, operators may interpret them as potential threats. This view is supported by Baethge et al. (37), who noted that when perceived responsibility exceeds an individual's coping capacity, it can become a threat stressor, thereby intensifying stress levels.

Conversely, responsibility pressure may also represent an opportunity for personal growth, learning, or achievement. Out of fear of being held accountable, operators may proactively improve their skills or heighten their concentration in order to prevent accidents. In this regard, responsibility pressure may be appraised as a challenge. This interpretation is consistent with Van de Poel and Sand (38), who emphasized that accountability and responsibility are central to fostering responsible innovation. Therefore, based on above discussion, we formulate the following hypotheses:

*H9. Responsibility pressure is positively associated with threat appraisal*
*H10. Responsibility pressure is positively associated with challenge appraisal*

### 2.6 Threat appraisal, emotion-focused coping and safety behavior

Although the relationship among threat appraisal, emotion-focused coping, and safety behavior has been examined in other domains, such as information security (39) and construction work (40), the findings remain inconclusive. For instance, Liang et al. (41) demonstrated that emotion-focused coping mediates the relationship between threat appraisal and safety behavior in the context of IT. In contrast, Krok et al. (42) found that this mediating role of emotion-focused coping was not observed in the associations between threat appraisal and survivors' health behavior among survivors during the COVID-19 pandemic. These inconsistencies underscore the importance of investigating these relationships in new, context-specific settings, such as special equipment operation, which is an area that remains underexplored.

Besides, in high-risk and high-stress occupations like special equipment operation, understanding how individuals appraise threats and respond emotionally is critical for promoting safe work practices. Prior studies have shown that threat appraisal is significantly related to the adoption of emotion-focused coping strategies in high-stress environments (43), and emotional regulation can contribute to safety behaviors (44). That is, threat appraisal may be associated with safety behaviors through emotion-focused coping strategies. Guided by the TTS proposed by Lazarus and Folkman (13), this study seeks to clarify the psychological mechanisms linking threat appraisals to safety behavior. Specifically, it explores whether emotion-focused coping serves as a mediator in this relationship. Thus, we formulate the following hypothesis:

*H11. Emotion-focused coping mediates the influence of threat appraisal on safety behavior*

### 2.7 Harm appraisal, emotion-focused coping and safety behavior

When operators encounter hazardous environments or recall past accidents, they may interpret these experiences as indicators of irreversible harm, triggering emotional distress such as anxiety, fear, or helplessness (45, 46). In response to such harm appraisals, individuals are more likely to adopt emotion-focused coping strategies, which aim to regulate internal emotional states. These strategies may include avoidance, denial, self-blame, or emotional venting (47). Although emotion-focused coping can offer temporary psychological relief, prior research suggests that in high-risk operational contexts, such strategies are often ineffective, as they do not address the root causes of the stressor or promote safety compliance (48–50). Accordingly, emotion-focused coping may appear to have limited association with on the relationship between harm appraisal and safety behavior.

However, based on the Transactional Theory of Stress (TTS), it is also plausible that emotion-focused coping serves as a mediator in this relationship. Specifically, when harm is appraised, operators may engage in emotionally driven coping behaviors, such as talking with friends or participating in leisure activities, to reduce negative emotional responses and reframe their perception of harm. This form of emotion regulation may help restore a sense of control and is associated with an increased capacity to engage in safe behaviors. Therefore, harm appraisal may be indirectly associated with safety behavior through the mediating role of emotion-focused coping. As mentioned above, we formulate the following hypothesis:

*H12. Emotion-focused coping mediates the influence of harm appraisal on safety behavior*

### 2.8 Challenge appraisal, problem-focused coping and safety behavior

Lee et al. (51) found that problem-focused coping strategies significantly improved safety compliance in the manufacturing industry, resulting in a 15% reduction in workplace accidents. These findings suggest that problem-focused coping plays a crucial role in enhancing workplace safety. Given that the special equipment industry is classified as a high-risk sector, it is particularly important to identify the role of problem-focused coping in improving safety behavior.

Current research has discussed the mediator role of problem-focused coping in the relationship between Challenge appraisal and safety sector in different aspects. For example, Kim et al. (52) stated that workers in high-risk industries, who appraised stressful situations as challenges, were more likely to adopt problem-solving strategies. This appraisal process enhances coping efficacy, leading to improved safety behavior and reduced risk of accidents. Similarly, Gonzalez and Morris (53) provides evidence that challenge appraisal is a key predictor of problem-focused coping in high-stress occupations. Their research demonstrates that employees who perceive workplace stressors as challenges are more likely to adopt proactive coping strategies, which in turn enhances their adherence to safety measures. These findings support the notion that problem-focused coping serves as a mediating mechanism between challenge appraisal and safety behavior, thereby promoting safer work practices. However, there is a limitation of empirical research identity the mediator of problem-focused coping in the relationship between Challenge appraisal and safety behavior in special equipment industry. Thus, the following hypothesis is proposed:

*H13. Problem-focused coping mediates the influence of challenge appraisal on safety behavior*

### 2.9 Government intervention, emotion-focused coping and safety behavior

Government intervention refers to any legal action undertaken by a governing authority aimed at influencing public decisions to enhance the social welfare of a nation (54). It takes many forms, including administrative and judicial monitoring, and is designed to ensure that controlled companies adhere to set norms and standards (55). In recent years, as global attention to occupational safety and health has increased, government intervention has been recognized as a key external control mechanism in ensuring operators' safety behavior (4). This is particularly critical in high-risk industries such as construction, manufacturing and mining, where government intervention is seen as a vital factor in maintaining operational safety (56). Wang et al. (49, 50) found that government supervision in China's mining industry provided operators with clear behavioral guidelines, which are associated with accident prevention and improved overall safety performance. Similarly, in the special equipment industry, the Chinese government has introduced relevant laws and regulations (such as Measures for the Supervision and Management of Personnel Operating Special Equipment) aimed at reducing accident rates and ensuring operators adhere to safety protocols.

However, although the government has promulgated these regulations, accidents continue to occur annually, raising questions about whether the current regulatory approach should evolve in response to social development. This raises critical questions about whether existing regulatory approaches are sufficient or need to evolve in response to societal and industrial changes. More importantly, government intervention may have dual effects. On one hand, it can induce stress or negative emotions among operators, potentially affecting their wellbeing (57). On the other hand, safety campaigns, training mandates, and inspection systems can raise safety awareness, foster positive safety attitudes, and encourage individuals to channel their emotion-focused coping strategies into more constructive and compliant behaviors. Therefore, based on the above discussion, we propose the following hypothesis:

*H14: Government intervention moderates the relationship between emotion-focused coping and safety behavior*

## 3 Research methodology

### 3.1 Research design

This study employed quantitative methods to collect data, with the goal of providing a more comprehensive and accurate understanding of the associations between stressors and individuals' behavior. The target sample for this study consisted of special equipment operators who has the at least 1 year working experience in China, to ensure sample diversity and statistical power. Furthermore, for ethical reasons, all volunteers are required to sign an informed consent form to confirm their comprehensive understanding of the experiment's objective, methods, and potential dangers.

### 3.2 Questionnaire design

#### 3.2.1 Survey, scale and consent form

A self-administered questionnaire was used to collect data, all measurement items were adapted from previous studies (8, 30, 36, 58–61), with detailed information on the items and their sources provided in Appendix 1. The demographics section included questions about the individual, such as age, kinds of operators and education. The second section of variables, respondents indicated the degree to which they agreed with statements using a five Likert scale (1 = strongly disagree, 3 = neutral, 5 = strongly agree), a widely used approach in studies based on the transactional theory of stress (8). The questionnaire distribution was examined and sanctioned by China Jiliang University, accompanied by an officially stamped informed consent form from the university. The informed consent form encompasses the following key elements: (1) a detailed explanation of the purpose and procedure of the questionnaire distribution; (2) a declaration that the researchers have not identified any foreseeable risks associated with participation in the study; (3) an assurance regarding the confidentiality and protection of participants' personal information; (4) a statement affirming that participation is entirely voluntary and that respondents may withdraw from the study at any point without any penalty; and (5) additional essential information pertaining to the questionnaire-based research.

#### 3.2.2 Data collection

Due to Chinese special equipment operators are a special group work in a complex environment, which is generally difficult to access. While Chinese special equipment operators demonstrate a high degree of obedience to their superiors, a top-down method was used to collect survey data, being more appropriate for data collection in China (30). The top-down approach required researcher to contact the director of the relevant department of each special equipment company (both production and usage enterprises) and examination center. After obtaining permission from senior management, the questionnaire link distributed to the director of different special equipment enterprises (both production and usage enterprises) and examination center via WeChat (The sample comprised 11 organizations located across eastern, central, and western regions of China, representing areas with varying levels of economic development), the director would assist in ensuring the smooth process of collecting data. Meanwhile, snowball sampling was employed to effectively identify the target population and collect comprehensive, contextually relevant information (62). Also, to mitigate the introduces bias of snowball sampling, multiple referral chains were initiated across different regions and companies, and a relatively large sample size (*n* = 735) were collected. The questionnaire link was initially distributed to a group of special equipment operators, who were then encouraged to forward the link to other operators. This approach facilitated the dissemination of the questionnaire to the intended sample and enhanced data collection.

In order to ensure the reliability and validity, this study spend a longtime for data collection, the data were collected from October 2024 to May 2025. Also, to prevents missing data, all questions were mandatory, and a screening question was presented at the beginning of the survey: “Are you a special equipment worker (at least 1 year work experience)?” The questionnaire proceeded only if the respondent answered “Yes.” A total of 745 responses were collected. Before conducting the analysis, the data were carefully cleaned and screened for quality. Questionnaires with substantial missing data, inconsistent responses, or abnormally short response times were excluded from the final sample. After data cleaning, 735 valid responses remained and were deemed suitable for analysis, as models with above seven constructs should utilized sample sizes of approximately 500 (63). The details of the respondents' demographic profiles are shown in Table 1.

**Table 1:** Demographic profiles of the respondents.

**Characteristics**	**Frequency (*n*)**	**Percentage (%)**
**Kinds of operator**		
Boiler operator	85	11.6
Pressure vessel operator	40	5.4
Gas cylinder operator	61	8.3
Elevator operator	41	5.6
Passenger ropeway operator	71	9.7
Large amusement ride operator	60	8.2
Industrial vehicle operator	123	16.7
Safety accessories maintenance personnel	62	8.4
Welder	66	9
Safety manager	40	5.4
Crane operator	86	11.7
**Age**		
25 and below	58	7.9
26–35	204	27.8
36–45	231	31.4
46–55	171	23.3
56 and above	71	9.7
**Monthly income**		
Under 3,000 yuan	35	4.8
3,001–3,500 yuan	60	8.2
3,501–4,500 yuan	97	13.2
4,501–5,500 yuan	170	23.1
5,501–6,500 yuan	174	23.7
6,501 yuan and above	199	27.1
**Education level**		
Secondary school and lower	220	29.9
Diploma	173	23.5
Undergraduate	300	40.8
Postgraduate	42	5.7
Total	735	100

## 4 Data analysis and discussion

### 4.1 Data analysis

This work employed a variance-based partial least squares structural equation model (PLS-SEM), with data analysis conducted using Smart-PLS software version 4.1.0.3 (SmartPLS GmbH, Monheim am Rhein, Germany), which provides extensive computational and modeling capabilities, along with professional assistance (64). Additionally, PLM-SEM does not need any distribution assumptions, and maximizes the explanatory variance of the proposed model (65) and enables users to access more intricate models with many variables, indicator constructs, and structural routes (66).

Following the two-step approach, this study first assessed the measurement model, and then the structural model will be provided. To assess the measurement model, the confirmatory factor analysis (CFA) was used to examine internal consistency, and construct reliability and validity. Cronbach's α and compound reliability above 0.7 were used to estimate internal consistency and structural reliability (67). Convergent and discriminant validity were used to evaluate construct validity. To demonstrate convergent validity, the composite reliability (CR) must exceed 0.7, and the average variance extracted (AVE) should surpass 0.5 while being lower than the corresponding CR (68). In the second step, the bias-corrected bootstrapping approach is utilized to test the suggested structural model and assumptions through 5,000 repetitions. All tests in this study were two-tailed, and a *p*-value of less than 0.05 was statistically significant.

#### 4.1.1 Measurement model

Initially, this study evaluated the measurement model by implementing CFA. Table 2 shows the evaluation results of the measurement model. The factor loadings for all items were over 0.7 and statistically significant. For all the structures, Cronbach's alpha (ranging from 0.843 to 0.951) and Composite reliability (ranging from 0.894 to 0.962) all exceed 0.7, which indicates good internal consistency and construct reliability. In addition, the AVE of all structures is over 0.5 (ranging from 0.671 to 0.836), which supports the convergence validity.

**Table 2:** Measurement model assessment.

**Constructs**	**Outer loadings**	**Cronbach's alpha**	**Composite reliability**	**AVE**
Low social status (LSS)		0.908	0.931	0.730
	0.838			
	0.869			
	0.838			
	0.854			
	0.870			
Harsh working environment (HWE)		0.840	0.906	0.762
	0.894			
	0.900			
	0.823			
Physiological fatigue (PF)		0.922	0.945	0.811
	0.891			
	0.917			
	0.903			
	0.891			
Responsibility pressure (RP)		0.883	0.928	0.811
	0.892			
	0.902			
	0.907			
Threat appraisal (TA)		0.877	0.911	0.671
	0.760			
	0.828			
	0.809			
	0.849			
	0.847			
Harm appraisal (H)		0.895	0.9634	0.826
	0.897			
	0.924			
	0.906			
Challenge appraisal (CA)		0.875	0.923	0799
	0.884			
	0.904			
	0.894			
Emotion-focused coping (EFC)		0.823	0.894	0.739
	0.834			
	0.879			
	0.865			
Problem-focused coping (PFC)		0.921	0.944	0.808
	0.892			
	0.899			
	0.913			
	0.891			
Safety behavior (S)		0.951	0.962	0.836
	0.911			
	0.920			
	0.921			
	0.922			
	0.897			
Government intervention (G)		0.928	0.943	0.734
	0.803			
	0.861			
	0.885			
	0.861			
	0.877			
	0.852			

Harman's Single Factor Test is a simplistic test to compute the common method variance (69). The study performed factor analysis using the principal axis factoring method with all the items in the model to verify whether one single factor will appear. In this study, Table A1 shows the common method variance resulted in 37.912% which is below the 50% threshold (70, 71), hence, indicating that the common method variance is not a considerable concern in this study. And the VIF values for all the items were below five, as seen in Table A2, which means that there are no collinearity issues.

In terms of discriminant validity, both the Fornell–Larcker criterion and HTMT were employed. The AVE for each construct exceeded the squared correlations between constructs, as seen in Tables A3, A4 shows that HTMT values less than the recommended threshold of 0.90, discriminant validity is still considered satisfactory (67, 72).

#### 4.1.2 Structural model

In this study, the coefficient of determination for the endogenous constructs or the dependent constructs. That is, CA (*R*^2^ = 0.125), EFC (*R*^2^ = 0.505), H (*R*^2^ = 0.340), PFC (*R*^2^ = 0.661), and S (*R*^2^ = 0.797), as seen in Table A5. It indicates that *R*^2^ of each endogenous variable meets the standard value (0–1), suggesting that the endogenous constructs are adequately explained by their respective predictors. Overall, these results demonstrate that the structural model exhibits satisfactory explanatory power and that the dependent constructs are predicted with a high degree of accuracy.

Besides, the predictive relevance of the model was evaluated using the *Q*^2^criterion. As shown in Table A6, all *Q*^2^values are greater than zero, providing evidence of satisfactory predictive relevance of the structural model.

Moreover, the reported effect sizes (*f*^2^; Table A7) indicate that CA was strong associated with TA (*f*^2^ = 1.952), while PFC showed a large association with H (*f*^2^ = 0.489). Moderate associations with sizes were observed for EFC on G (*f*^2^ = 0.212) and TA on G (*f*^2^ = 0.186). In contrast, RP showed small to moderate associations with CA (*f*^2^ = 0.108) and TA (*f*^2^ = 0.090), whereas H on G (*f*^2^ = 0.037), HWE on H (*f*^2^ = 0.032), PF on H (*f*^2^ = 0.009), LSS on G (*f*^2^ = 0.007), and HWE on G (*f*^2^ = 0.001) exhibited small or negligible associations. Overall, these findings suggest that CA and PFC are the strongly associated predictors in the model, while the remaining relationships contribute limited explanatory power to the endogenous constructs.

**Figure 3:**
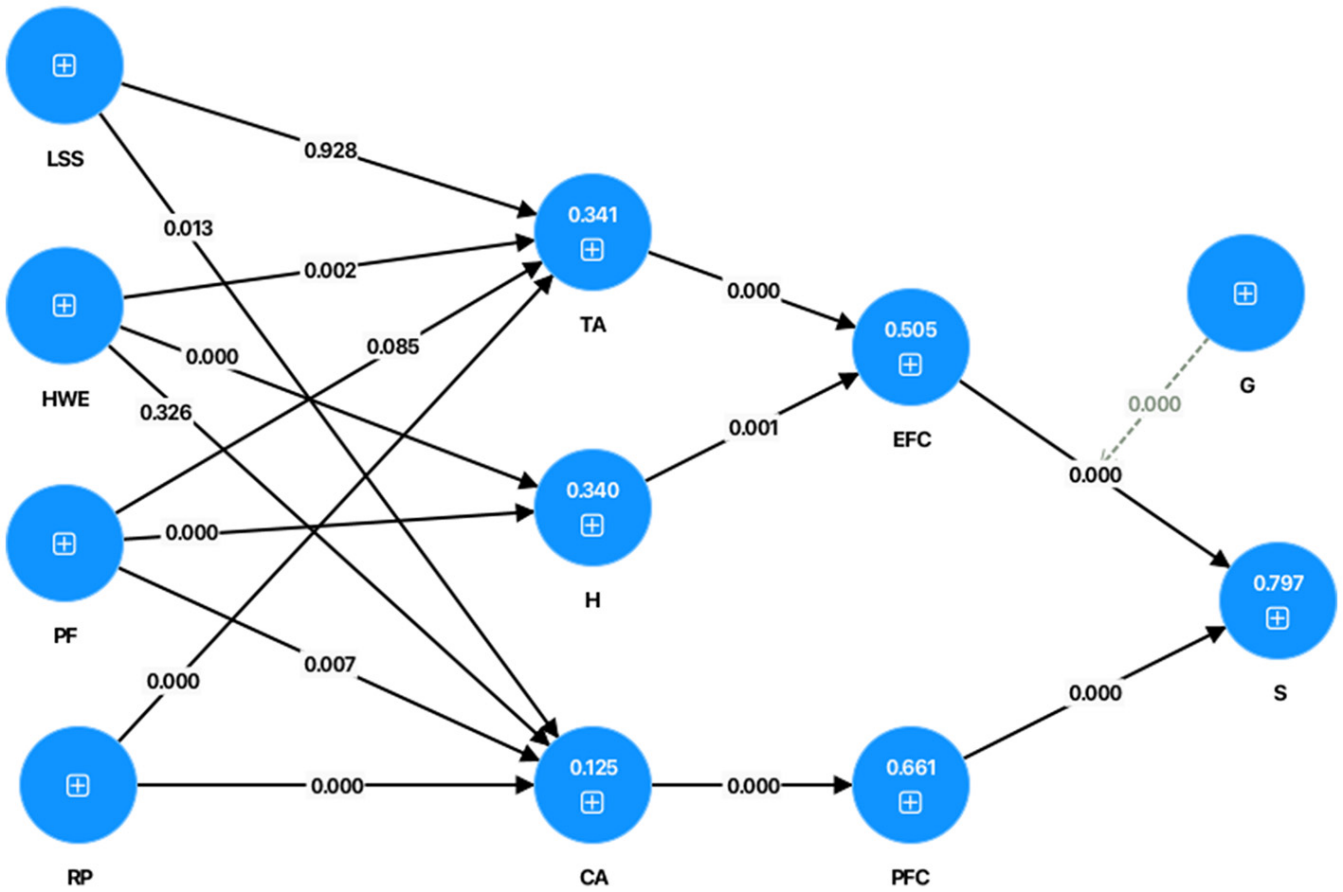
The result of structural model.

Furthermore, the results of the structural model analysis are presented in Tables 3, 4, and the proposed model is illustrated in Figure 3. Based on the standard thresholds for statistical significance (*p*-value < 0.05 and *T*-value >1.96), the hypothesis testing results indicate that eleven hypotheses (H2, H3, H4, H7, H8, H9, H10, H11, H12, H13, and H14) were supported, whereas four hypotheses (H1, H5, and H6) were not supported. In details, the structural model analysis and hypotheses tests explained that:

- H4, H7, H9, H10, H11, H13, and H14 were accepted as a significance level of 0.000. Hence HWE and PF were significantly positively associated with H with a β value of 0.238 and 0.377. RP was significantly positively associated with TA and CA with a β value of 0.355 and 0.448. EFC significate positive mediates the relationship between TA and S with a β value of 0.050. PFC significate positive mediates the relationship between CA and S with a β value of 0.436. G moderate the relationship between EFC and S with a β value of −0.081.
- H2 was accepted at a significance level of 0.01. Hence LSS had a significant negative associated with CA with a β value of −0.135.
- H3, H8, and H12 were accepted at a significance level of 0.001. Therefore, HWE had a significant positive associated with TA with a β value of 0.175. RF had a significant negative associated with CA with a β value of −0.178. EFC significate positive mediates the relationship between H and S with a β value of 0.022.
- H1, H5, and H6 were rejected, were not supported, as their corresponding *p*-values exceeded the 0.05 significance threshold. Specifically, LSS had no significant associated with CA with a *p*- value of 0.928. HWE had no significant associated with CA with a *p*-value of 0.326. PF didn't associated with TA with a *p*-value of 0.085, respectively.

**Table 3:** Path coefficients.

**Hypotheses**	**Path**	**Std. Beta (β)**	***T*-value**	***P*-value**	**Result**
H1	LSS → TA	0.004	0.091	0.928	Rejected
H2	LSS → CA	−0.135	2.488	0.013^*^	Supported
H3	HWE → TA	0.175	3.127	0.002^**^	Supported
H4	HWE → H	0.238	4.173	0.000^***^	Supported
H5	HWE → CA	0.059	0.983	0.326	Rejected
H6	PF → TA	0.109	1.721	0.085	Rejected
H7	PF → H	0.377	6.675	0.000^***^	Supported
H8	PF → CA	−0.178	2.703	0.007^*^	Supported
H9	RP → TA	0.355	7.113	0.000^***^	Supported
H10	RP → CA	0.448	10.188	0.000^***^	Supported
H14	G x EFC → S	−0.081	4.839	0.000^***^	Supported

^*^p < 0.05; ^**^p < 0.01; ^***^p < 0.001.

**Table 4:** Mediation effect.

**Hypotheses**	**Path (indirect effect)**	**Std. Beta (β)**	**95% confidence interval between indirect effect**	***T*-value**	***P*-value**	**Result**
H11	TA → EFC → S	0.050	(0.028, 0.077)	4.011	0.000	Supported
H12	H → EFC → S	0.022	(0.008, 0.040)	2.740	0.006	Supported
H13	CA → PFC → S	0.436	(0.370, 0.504)	12.596	0.000	Supported

## 5 Discussion

One of the aims of this study is to identify the stressors of special equipment operators that are associated with their safety behavior, based on the transactional theory of stress (TTS). The results indicate that low social status (LSS), harsh work environment (HWE), physiological fatigue (PF), and responsibility pressure (RP) are key stressors, as each is significantly associated with different types of appraisal (threat, harm, or challenge). Specifically, HWE and RP were strongly associated with threat appraisal (TA), HWE, and PF were strongly associated with harm appraisal (H), and LSS, PF, and RP were strongly associated with challenge appraisal (CA). To our knowledge, this is the first study to identify the stressors of Chinese special equipment operators.

HWE and PR were strongly positively associated with TA, it means that both HWE and PR will evoke operators' threat. In details, operator perceived the threat (like heathy threat) when facing the unchangeable harsh work environment, the worse harsh work environment is, the more threat they perceived. This result is consistent with the view from So et al. (14), that threat appraisal occurs when the situation is perceived as potentially harmful, posing risks to wellbeing or performance, leading to stress or anxiety. Also, the finding indicated that operator would receive much threat if the heaven responsibility pressure they get. This point is supported by Baethge et al. (37), when the perceived responsibility surpasses the operator's coping mechanisms, it may transition into a threat stressor, exacerbating stress levels.

HWE and PF were strongly positively associated with H, that is, HWE and PF will arouse operators' harm. Appraisal. In details, as the severity of the environment increases, operator will feel more harm. The result is supported by Lazarus and Folkman (13), that harm appraisal involves the recognition that damage has already occurred, whether physical, emotional, or in terms of performance. Besides, the finding also explained that operators' physiological fatigue will let them perceive more harm. This is supported by Xing et al. (34) indicated that the physiological fatigue can induce the mental fatigue status and other mental performances of the brain.

LSS, HWE, PF, and RP were strongly positively with CA, it indicates that low social status, hash working environment, physiological fatigue and responsibility pressure are perceived by operators as significant challenges. Low social status showed a negative association with challenge appraisal, suggesting that when operators perceive themselves as having a lower social status, they are less likely to interpret work-related demands as opportunities for growth or achievement. Instead, they may view these demands as burdens, which can reduce motivation and engagement in the workplace. Besides, the result suggests that operators may perceive a harsh working environment as a challenge, and this perceived challenge tends to intensify as the severity of the environment increases. In other words, the more demanding or uncomfortable the working conditions, the more likely operators are to appraise them as significant challenges, potentially impacting their behavioral responses. This is similar with Liang (29), who suggest that under certain conditions, employees may exhibit greater creativity and proactive behavior when confronting stress. Also, the finding illustrates the physiological fatigue is negatively associated with operators' challenge appraisal. This suggests that as operators become physically exhausted, they are less likely to view demanding tasks as opportunities for growth or achievement. Instead, fatigue may undermine their motivation and reduce their ability to engage positively with challenges, leading to lower performance and increased risk of errors in high-risk work environments. This view is supported by Zhang et al. (17), who found that construction workers' physical fatigue is negatively associated with cognitive and motor abilities. Moreover, responsibility pressure is positively associated with challenge appraisal. This suggests that when operators feel a strong sense of responsibility, they are more likely to interpret demanding tasks as meaningful challenges. The internal motivation driven by accountability may enhance their engagement, resilience, and willingness to overcome difficulties in high-pressure work environments. This finding is similar with Van de Poel and Sand (38), who emphasized that accountability and responsibility are central to fostering responsible innovation.

The results indicate that emotion-focused coping mediates the relationship between threat appraisal and safety behavior, as well as between harm appraisal and safety behavior. This suggests that when operators perceive a situation as threatening or harmful, their emotional coping strategies, such as seeking social support or trying to manage stress play a key role in shaping their subsequent safety behaviors. Emotion-focused coping may buffer the negative associations of these appraisals, potentially promoting more cautious or compliant safety behavior. These findings are consistent with the transactional theory of stress (TTS), but contrast with previous studies. Krok et al. (42) reported that the mediating role of emotion-focused coping was not observed in the relationship between threat appraisal and survivors' health behavior during the COVID-19 pandemic. Similarly, Searle and Lee (48) and Wang et al. (49), Wang et al. (50) found that emotion-focused coping is often less effective, as it does not address the underlying stressors or enhance safety compliance.

This study found that problem-focused coping was mediates the relationship between challenge appraisal and safety behavior. This study found that problem-focused coping mediates the relationship between challenge appraisal and safety behavior. This indicates that when operators perceive work demands as challenges, they are more likely to engage in proactive problem-solving strategies, which in turn promote safer behaviors. Problem-focused coping may function as an important psychological mechanism through which positive cognitive appraisals are related to increased safety behaviors, acknowledging that causality cannot be established.

Finally, Government intervention negative moderate the relationship between emotion-focused coping and safety behavior. These findings indicate that under conditions of strong or intrusive government intervention, the positive association between emotion-focused coping and safety behavior appears weaker. In such cases, operators who rely on emotional regulation strategies, such as stress relief or emotional support may feel that external control undermines their personal coping efforts, thereby reducing their engagement in safe practices. This is consistent with Ford and Feinberg (57), that government intervention may induce stress or negative emotions among operators, potentially affecting their wellbeing.

## 6 Conclusion

### 6.1 Theoretical implications

To authors' knowledge, this is the first academic study focusing on the special equipment industry in China, with particular emphasis on the stressors, psychological states, and behaviors of special equipment operators. The findings of this research will serve as a valuable reference for other scholars interested in the special equipment industry or the wellbeing and performance of special equipment operators.

Besides, this study enriches and extends the application of the Transactional Theory of Stress (TTS) within the context of special equipment operators in China. By examining multiple stressors of low social status, harsh working environments, physiological fatigue, and responsibility pressure, it addresses a gap in the literature regarding how these stressors are associated with operators' appraisals, coping strategies, and subsequent safety behaviors. The developed model demonstrated strong predictive power in explaining the safety behaviors of special equipment operators under various stress conditions.

Moreover, the research provides novel insights into the mediating roles of emotion-focused coping between threat appraisal or harm appraisal and safety behavior, as well as problem-focused coping between challenge appraisal and safety behavior in the sector of special equipment operator, thereby deepening theoretical understanding of coping mechanisms in high-risk occupational settings.

Finally, by incorporating government intervention as a moderating variable, this study highlights how external environmental factors are associated with the relationship between coping strategies and safety behaviors, thereby expanding the scope of transactional stress theory and offering new theoretical perspectives for future research.

### 6.2 Practical implications

This study offers several practical implications for industry practitioners and policymakers in the special equipment sector. First, by identifying key stressors such as low social status, harsh working environments, physiological fatigue, and responsibility pressure, organizations and government can develop targeted interventions to alleviate these pressures, thereby enhancing operators' psychological wellbeing and safety performance. For example, organizations can reasonably arrange work and rest schedules to ensure that operators are not subjected to prolonged periods of fatigue, thereby reducing their threat perception. Moreover, given the critical role of operators in the special equipment industry, government authorities could consider adopting professional title recognition and qualification systems, similar to the certification framework for construction engineers, to enhance the social status of these workers. Such measures can help alleviate stress caused by perceived low social standing and foster a more motivated and psychologically healthy workforce.

Second, the findings emphasize the importance of fostering effective coping strategies, particularly problem-focused coping and emotion-focused coping. Organizations should provide training and resources that help operators develop these coping mechanisms. For example, promoting problem solving skills, encouraging proactive communication, and offering stress management workshops can empower operators to handle work-related challenges more effectively. At the same time, emotional support systems, such as peer support groups, mental health hotlines, or access to counseling services can strengthen emotion-focused coping, helping operators regulate their emotional responses to stress. Together, these strategies contribute to improved safety behavior and overall wellbeing in high-risk working environments.

Third, the moderating role of government intervention suggests that policymakers should carefully design regulations and support systems that empower operators without undermining their personal coping efforts. Balanced and context-sensitive government policies can promote a safer and more supportive working environment. For instance, enforce reasonable labor standards, including limits on overtime. Or launch public welfare campaigns to improve operators' public image and social recognition.

### 6.3 Limitations and future research

First, this study focused on four specific stressors of low social status, harsh working environment, physiological fatigue, and responsibility pressure. However, other relevant stressors may also exist. Future research could adopt qualitative methods to explore additional or emerging stressors affecting special equipment operators.

Second, this study treated operators as a homogeneous group. While this approach provides generalizable insights, the category of special equipment operators includes 11 different types of roles. Future studies could conduct subgroup analyses for each category to generate more targeted and actionable findings.

Third, this research examined government intervention only as a moderating variable between coping strategies and safety behavior. Future studies could further explore the role of government intervention at various stages of the stress-coping-safety behavior process, helping to identify where regulation is most effective or lacking. Additionally, government intervention encompasses multiple dimensions. For example, legally enforced restrictions vs. mechanisms for protecting workers' rights. These different forms may be associated with varying outcomes, and future studies should consider disaggregating and analyzing their individual impacts.

## Data Availability

The original contributions presented in the study are included in the article/Supplementary material, further inquiries can be directed to the corresponding author.

## Footnotes

1 Special equipment is categorized into two main types: pressure-bearing special equipment, which includes boilers, pressure vessels (such as gas cylinders), and pressure pipelines, and electromechanical special equipment, encompassing elevators, lifting machinery, passenger ropeways, large amusement facilities, and special motor vehicles used within premises (factories).

2 Special equipment operators can generally be classified into two categories. The first category includes personnel responsible for the operation of boilers, pressure vessels, gas cylinders, elevators, lifting machinery, passenger ropeways, large amusement facilities, and industrial vehicle operator, as well as safety accessory maintenance personnel and welders engaged in special equipment-related tasks. The second category consists of safety manager for supervising and overseeing the above-mentioned operations.
