# Supplementary material for: Understanding the impact of stressors on safety behavior of Chinese special equipment operators: a transactional theory of stress perspective
Source: Front Public Health. 2026 Mar 4;14:1775181. doi: 10.3389/fpubh.2026.1775181 (PMC12996098; doi:10.3389/fpubh.2026.1775181)
Supplement: Supplementary file 1 [file Data_Sheet_1.docx]

**Appendix 1:**

**Section A: Demographic Research**

Please read each question carefully and provide the correct information by placing a TICK (√) in the boxes provided.

1. Are you a special equipment operator with at least one year of relevant work experience?

|  | Yes |
| --- | --- |
|  | Not |

1. Which type of special equipment operator do you belong to?

|  | Boiler Operator |
| --- | --- |
|  | Pressure Vessel Operator |
|  | Gas Cylinder Operator |
|  | Elevator Operator |
|  | Passenger Ropeway Operator |
|  | Large Amusement Ride Operator |
|  | Industrial Vehicle Operator |
|  | Safety Accessories Maintenance Personnel |
|  | Welder |
|  | Safety Manager |
|  | Crane Operator |

1. What is your age?

|  | 25 and under |
| --- | --- |
|  | 26-35 |
|  | 36-45 |
|  | 46-55 |
|  | 56 over |

1. What is your monthly income?

|  | 3000 or less |
| --- | --- |
|  | 3001-3500 |
|  | 3501-4500 |
|  | 4501-5500 |
|  | 5501-6500 |
|  | 6501 and above |

1. What is your education level?

|  | High school (including technical secondary school) and lower |
| --- | --- |
|  | College degree |
|  | Graduate degree |
|  | Postgraduate degree or higher |

**Section B**

Please TICK (√) the following choices provided to indicate your level of agreement or disagreement with the statements posed.

| Strongly Disagree | Disagree | Neutral | Agree | Strongly Agree |
| --- | --- | --- | --- | --- |
| 1 | 2 | 3 | 4 | 5 |

| **Constructs** | **Items** | **Citation** |
| --- | --- | --- |
| Low social status |  |  |
|  | My family feels embarrassed about my job in the special equipment industry. | Wan et al. (2014) |
|  | Special equipment operator is not a respected occupation. |  |
|  | Special equipment operator is not an important job in our society. |  |
|  | Special equipment operator is not valuable to the society. |  |
|  | I feel inferior about my career in the special equipment. |  |
| Harsh work environment |  |  |
|  | I do not feel safe in a harsh work environment. | Duffy et al. (2017) |
|  | Harsh working conditions make me feel afraid at work. |  |
|  | I lack a sense of security at work. |  |
| Physiological fatigue |  |  |
|  | I am yawning at work. | Gu & Guo (2022) |
|  | I am drowsy at work. |  |
|  | I have muscle stiffness at work. |  |
|  | I feel eye strain at work. |  |
| Responsibility pressure |  |  |
|  | Working with special equipment requires me to bear considerable responsibility, which makes me feel very stressed. | Jiandong et al. (2022) |
|  | If my work leads to a safety accident, I will bear considerable responsibility, which indirectly increases my stress. |  |
|  | Working as a special equipment operator involves a wide range of job responsibilities, which makes me feel very stressed. |  |
| Threat appraisal |  |  |
|  | I feel that the responsibility pressure of operating special equipment puts me at risk of significant consequences, as even small errors could lead to severe financial or safety impacts. | Sharma & Gupta (2023);  So et al. (2016) |
|  | I worry that the harsh work environment, combined with the demands of operating special equipment, significantly increases the risk of accidents or physical strain. |  |
|  | I feel that the low social status associated with being a special equipment operator means that my concerns about safety and operational risks are often undervalued or ignored. |  |
|  | The pressure from role overload makes me feel overwhelmed, and I fear that the constant demands of operating special equipment will lead to burnout or critical mistakes. |  |
|  | Financial insecurity adds to the stress I experience while operating special equipment, making it harder to maintain focus and increasing the risk of costly errors or inefficiencies. |  |
| Harm appraisal |  |  |
|  | Due to the physical fatigue from operating special equipment for extended periods, I am unable to maintain the level of performance I would expect from myself. | Sharma and Gupta (2023) |
|  | I have had to compromise significantly on my physical health because the harsh conditions under which I operate special equipment are exhausting and physically taxing. |  |
|  | The persistent physical strain and demanding environment associated with operating special equipment have had a clear negative effect on both my health and my ability to perform at my best. |  |
| Challenge appraisal |  |  |
|  | I see the responsibility pressure of operating special equipment as an opportunity to sharpen my decision-making skills, knowing that even small mistakes can have significant consequences. | Sharma & Gupta (2023) |
|  | I view the responsibility pressure of operating special equipment as a chance to build resilience, as the demanding tasks require me to stay focused and maintain high performance under stress. |  |
|  | I feel that I am successfully handling the responsibility pressure of operating special equipment, using it as a way to improve my professional skills and confidence in managing high-risk situations. |  |
| Emotion-focused coping |  |  |
|  | I actively seek emotional support from friends and family to help me deal with the stress and fear of making mistakes or causing accidents while operating special equipment. | Sharma & Gupta (2023) |
|  | I discuss with someone the physical and emotional toll that operating special equipment takes on me, especially when I feel it may be harming my health or safety. |  |
|  | I share my anxieties with others about the risks and potential harm involved in operating special equipment, particularly when I feel overwhelmed by the responsibility and potential dangers. |  |
| Problem-focused coping |  |  |
|  | I feel competent in tackling the challenges that come with operating special equipment, viewing them as valuable opportunities to improve my technical expertise and problem-solving abilities. | Sharma & Gupta (2023) |
|  | I feel confident in my ability to overcome the difficulties of operating special equipment, understanding that each challenge helps me refine my skills and become more efficient. |  |
|  | I feel hopeful that by directly addressing the challenges of operating special equipment, I can develop new skills and successfully handle increasingly complex tasks. |  |
|  | I feel motivated to proactively engage with the challenges of operating special equipment, knowing that overcoming these obstacles will enhance both my performance and technical expertise. |  |
| Safety behavior |  |  |
|  | I always wear the required personal protective equipment (PPE) when operating special equipment. | Cheng et al. (2020) |
|  | I strictly follow the safety operation procedures when operating special equipment. |  |
|  | I thoroughly check the special equipment and the work area before beginning operation. |  |
|  | I proactively cooperate with safety inspections related to the operation of special equipment. |  |
|  | I actively cooperate with the instructions from production safety control staff during equipment operation. |  |
| Government intervention |  |  |
|  | Government regulations strictly influence the safety standards for operating special equipment in company, ensuring that all operators consistently wear protective gear and follow safety procedures. | Gao (2011) |
|  | Company’s investment in special equipment is heavily shaped by government safety policies, which requires to prioritize the acquisition of equipment that enhances operator safety and reduces workplace hazards. |  |
|  | Government intervention guide company’s decisions, ensuring that they purchase special equipment that meets rigorous safety standards to protect employees. |  |
|  | Government labor and safety regulations directly influence company’s employment policies, ensuring that special equipment operators receive comprehensive safety training and are required to follow strict safety protocols. |  |
|  | Government intervention has greatly enhanced special equipment operators’ safety assurance. |  |
|  | Government safety and environmental regulations strongly influence company’s production decisions, enforcing the implementation of strict safety practices when operating special equipment to minimize risks. |  |

**Appendix 2 Table 3. The Total Variance**

| Component | Initial Eigenvalues | | | Extraction Sums of Squared Loadings | | |
| --- | --- | --- | --- | --- | --- | --- |
|  | Total | % of Variance | Cumulative % | Total | % of Variance | Cumulative % |
| 1 | 16.681 | 37.912 | 37.912 | 16.681 | **37.912** | 37.912 |
| 2 | 9.498 | 21.587 | 59.498 | 9.498 | 21.587 | 59.498 |
| 3 | 2.597 | 5.903 | 65.401 | 2.597 | 5.903 | 65.401 |
| 4 | 1.364 | 3.100 | 68.501 | 1.364 | 3.100 | 68.501 |
| 5 | 0.962 | 2.186 | 70.687 |  |  |  |
| 6 | 0.733 | 1.666 | 72.352 |  |  |  |
| 7 | 0.687 | 1.562 | 73.915 |  |  |  |
| 8 | 0.623 | 1.417 | 75.331 |  |  |  |
| 9 | 0.592 | 1.345 | 76.677 |  |  |  |
| 10 | 0.561 | 1.276 | 77.952 |  |  |  |
| 11 | 0.502 | 1.141 | 79.093 |  |  |  |
| 12 | 0.467 | 1.060 | 80.153 |  |  |  |
| 13 | 0.440 | 0.999 | 81.153 |  |  |  |
| 14 | 0.427 | 0.971 | 82.124 |  |  |  |
| 15 | 0.416 | 0.945 | 83.068 |  |  |  |
| 16 | 0.400 | 0.910 | 83.978 |  |  |  |
| 17 | 0.379 | 0.862 | 84.840 |  |  |  |
| 18 | 0.364 | 0.827 | 85.667 |  |  |  |
| 19 | 0.358 | 0.813 | 86.479 |  |  |  |
| 20 | 0.343 | 0.780 | 87.259 |  |  |  |
| 21 | 0.336 | 0.763 | 88.023 |  |  |  |
| 22 | 0.330 | 0.750 | 88.772 |  |  |  |
| 23 | 0.309 | 0.702 | 89.474 |  |  |  |
| 24 | 0.297 | 0.675 | 90.149 |  |  |  |
| 25 | 0.283 | 0.644 | 90.793 |  |  |  |
| 26 | 0.276 | 0.627 | 91.420 |  |  |  |
| 27 | 0.273 | 0.621 | 92.040 |  |  |  |
| 28 | 0.267 | 0.607 | 92.647 |  |  |  |
| 29 | 0.260 | 0.591 | 93.238 |  |  |  |
| 30 | 0.255 | 0.580 | 93.817 |  |  |  |
| 31 | 0.246 | 0.559 | 94.376 |  |  |  |
| 32 | 0.237 | 0.539 | 94.915 |  |  |  |
| 33 | 0.228 | 0.518 | 95.433 |  |  |  |
| 34 | 0.223 | 0.507 | 95.940 |  |  |  |
| 35 | 0.210 | 0.477 | 96.417 |  |  |  |
| 36 | 0.204 | 0.464 | 96.881 |  |  |  |
| 37 | 0.197 | 0.448 | 97.330 |  |  |  |
| 38 | 0.188 | 0.427 | 97.757 |  |  |  |
| 39 | 0.183 | 0.415 | 98.172 |  |  |  |
| 40 | 0.176 | 0.400 | 98.572 |  |  |  |
| 41 | 0.168 | 0.382 | 98.954 |  |  |  |
| 42 | 0.161 | 0.366 | 99.320 |  |  |  |
| 43 | 0.159 | 0.362 | 99.682 |  |  |  |
| 44 | 0.140 | 0.318 | 100.000 |  |  |  |
| Extraction Method: Principal Component Analysis. | | | | | | |

**Table 4. Variance inflation factor (Outer VIF)**

| **Constructs** | **Items** | **VIF** |
| --- | --- | --- |
| Challenge appraisal | CA1 | 2.254 |
|  | CA2 | 2.484 |
|  | CA3 | 2.358 |
| Emotion-focused coping | EFC1 | 1.663 |
|  | EFC2 | 2.072 |
|  | EFC3 | 1.964 |
| Government intervention | G1 | 2.440 |
|  | G2 | 3.220 |
|  | G3 | 3.339 |
|  | G4 | 2.783 |
|  | G5 | 3.152 |
|  | G6 | 2.636 |
| Harm appraisal | H1 | 2.486 |
|  | H2 | 3.064 |
|  | H3 | 2.693 |
| Harsh working environment | HWE1 | 2.293 |
|  | HWE2 | 2.436 |
|  | HWE3 | 1.705 |
| Low social status | LSS1 | 2.269 |
|  | LSS2 | 2.386 |
|  | LSS3 | 2.403 |
|  | LSS4 | 2.799 |
|  | LSS5 | 2.865 |
| Physiological fatigue | PF1 | 2.937 |
|  | PF2 | 3.700 |
|  | PF3 | 3.194 |
|  | PF4 | 2.797 |
| Problem-focused coping | PFC1 | 2.895 |
|  | PFC2 | 3.013 |
|  | PFC3 | 3.431 |
|  | PFC4 | 2.908 |
| Responsibility pressure | RP1 | 2.379 |
|  | RP2 | 2.530 |
|  | RP3 | 2.561 |
| Safety behavior | S1 | 3.837 |
|  | S2 | 4.279 |
|  | S3 | 4.246 |
|  | S4 | 4.401 |
|  | S5 | 3.425 |
| Threat appraisal | TA1 | 1.794 |
|  | TA2 | 2.140 |
|  | TA3 | 2.052 |
|  | TA4 | 2.359 |
|  | TA5 | 2.360 |

**Table 5. Discriminant validity assessment using Fornell-Larcker criterion**

|  | CA | EFC | G | H | HWE | LSS | PF | PFC | RP | S | TA |
| --- | --- | --- | --- | --- | --- | --- | --- | --- | --- | --- | --- |
| CA | 0.894 |  |  |  |  |  |  |  |  |  |  |
| EFC | 0.643 | 0.859 |  |  |  |  |  |  |  |  |  |
| G | 0.700 | 0.459 | 0.857 |  |  |  |  |  |  |  |  |
| H | 0.406 | 0.643 | 0.288 | 0.909 |  |  |  |  |  |  |  |
| HWE | 0.133 | 0.352 | 0.218 | 0.535 | 0.873 |  |  |  |  |  |  |
| LSS | 0.017 | 0.284 | 0.054 | 0.456 | 0.711 | 0.854 |  |  |  |  |  |
| PF | 0.069 | 0.356 | 0.116 | 0.564 | 0.789 | 0.788 | 0.901 |  |  |  |  |
| PFC | 0.813 | 0.619 | 0.733 | 0.354 | 0.064 | -0.015 | 0.010 | 0.899 |  |  |  |
| RP | 0.293 | 0.424 | 0.399 | 0.534 | 0.692 | 0.558 | 0.683 | 0.266 | 0.900 |  |  |
| S | 0.794 | 0.577 | 0.784 | 0.372 | 0.090 | -0.070 | 0.005 | 0.857 | 0.304 | 0.914 |  |
| TA | 0.525 | 0.698 | 0.447 | 0.806 | 0.509 | 0.412 | 0.493 | 0.459 | 0.553 | 0.488 | 0.819 |

**Table 6. Discriminant validity assessment using HTMT**

|  | CA | EFC | G | H | HWE | LSS | PF | PFC | RP | S | TA |
| --- | --- | --- | --- | --- | --- | --- | --- | --- | --- | --- | --- |
| CA |  |  |  |  |  |  |  |  |  |  |  |
| EFC | 0.758 |  |  |  |  |  |  |  |  |  |  |
| G | 0.771 | 0.519 |  |  |  |  |  |  |  |  |  |
| H | 0.459 | 0.749 | 0.313 |  |  |  |  |  |  |  |  |
| HWE | 0.158 | 0.421 | 0.251 | 0.615 |  |  |  |  |  |  |  |
| LSS | 0.053 | 0.324 | 0.092 | 0.502 | 0.818 |  |  |  |  |  |  |
| PF | 0.080 | 0.408 | 0.131 | 0.621 | 0.900 | 0.862 |  |  |  |  |  |
| PFC | 0.898 | 0.712 | 0.789 | 0.389 | 0.113 | 0.055 | 0.028 |  |  |  |  |
| RP | 0.333 | 0.497 | 0.447 | 0.600 | 0.802 | 0.615 | 0.756 | 0.296 |  |  |  |
| S | 0.871 | 0.652 | 0.829 | 0.403 | 0.133 | 0.104 | 0.026 | 0.890 | 0.332 |  |  |
| TA | 0.602 | 0.820 | 0.494 | 0.889 | 0.588 | 0.451 | 0.544 | 0.513 | 0.627 | 0.537 |  |

**Table 7. Coefficient of Determination (R^2^)**

|  | R Square |
| --- | --- |
| CA | 0.125 |
| EFC | 0.505 |
| H | 0.340 |
| PFC | 0.661 |
| S | 0.797 |

**Table 8. Validated Redundancy (Q^2^)**

|  | Q² (=1-SSE/SSO) |
| --- | --- |
| TA | 0.225 |
| H | 0.278 |
| CA | 0.095 |
| EFC | 0.370 |
| PFC | 0.531 |
| S | 0.662 |

**Table 9. Effect size ( f^2^)**

|  | CA | EFC | G | H | HWE | LSS | PF | PFC | RP | S | TA |
| --- | --- | --- | --- | --- | --- | --- | --- | --- | --- | --- | --- |
| CA |  |  |  |  |  |  |  | 1.952 |  |  |  |
| EFC |  |  |  |  |  |  |  |  |  | 0.027 |  |
| G |  |  |  |  |  |  |  |  |  | 0.212 |  |
| H |  | 0.037 |  |  |  |  |  |  |  |  |  |
| HWE | 0.001 |  |  | 0.032 |  |  |  |  |  |  | 0.015 |
| LSS | 0.007 |  |  |  |  |  |  |  |  |  | 0.000 |
| PF | 0.009 |  |  | 0.081 |  |  |  |  |  |  | 0.004 |
| PFC |  |  |  |  |  |  |  |  |  | 0.489 |  |
| RP | 0.108 |  |  |  |  |  |  |  |  |  | 0.090 |
| S |  |  |  |  |  |  |  |  |  |  |  |
| TA |  | 0.186 |  |  |  |  |  |  |  |  |  |
